# M. Donné on the Composition of Urine during Pregnancy and Disease

**Published:** 1842-04-30

**Authors:** 


*On the composition of the urine during Pregnancy and Disease.* By M. Donné.—M. Donné found that during pregnancy the urine possesses less uric acid and phosphate of lime than during the ordinary state of the body; a circumstance which it is easy to account for, seeing that these elements are required for the formation of the bones of the fœtus. The crystallization of the salts of the urine is then modified in such a remarkable manner that M. Donné has been able, by the inspection of them alone, to ascertain the existence of pregnancy in no fewer than thirty cases.

When a person is in good health, the urine contains iron; but during chlorosis iron is not to be detected by the usual reagents, but it may again be recognised if ferruginous preparations are administered. In certain forms of chlorosis he found that the urine contains iron, and in such cases ferruginous preparations are useless as a means of cure. The examination of the urine is, therefore, of considerable importance in all such cases It is stated that we never can be sure that the chlorotic affection is permanently removed, unless we ascertain by an examination of the urine several days after the ferruginous preparations are omitted, that iron is still passed off with it.

In typhoid fever the crystals of the urine presented a rayed aspect, and a pearly lustre, something like crystals of the phosphate of ammonia. The same appearance was observed during pneumonia.

M. Donné mentions that he has injected milk into the serous cavities of dogs, and also into the veins, which, provided it was pure, gave rise to no bad symptom, but circulated it in the vessels like blood.—*Ed. Med. and Surg. Journ.*, from *Comptes Rendus de l'Acad. des Sci.* May 24, 1841.

